# Measuring Precipitation via Microwave Bands with a High-Accuracy Setup

**DOI:** 10.3390/s24248056

**Published:** 2024-12-17

**Authors:** Alexandros Sakkas, Vasilis Christofilakis, Christos J. Lolis, Spyridon K. Chronopoulos, Kostas P. Peppas

**Affiliations:** 1 Electronics-Telecommunications and Applications Laboratory, Physics Department, University of Ioannina, 45110 Ioannina, Greece; a.sakkas@uoi.gr; 2 Laboratory of Meteorology, Physics Department, University of Ioannina, 45110 Ioannina, Greece; chlolis@uoi.gr; 3 Department of Informatics and Telecommunications, University of Peloponnese, Acadimaikou G.K. Vlachou, 22100 Tripolis, Greece; peppas@uop.gr

**Keywords:** experimental setup, signal attenuation, precipitation, microwave, measurements

## Abstract

The urgent need for timely and accurate precipitation estimations in the face of ongoing climate change and the increasing frequency and/or intensity of extreme weather events underscores the necessity for innovative approaches. Recently, several studies have focused on estimating the precipitation rate through induced attenuation of radio frequency (RF) signals, which are abundant in modern communication systems. Most research has concentrated on frequencies exceeding 10 GHz, as attenuation at lower frequencies is minimal, posing measurement challenges. This study aims to confront this limitation by introducing a high-precision experimental setup capable of detecting this subtle attenuation at frequencies under 10 GHz. The setup includes a transmitter and receiver optimized for operation at 2.07, 4.63, and 6.22 GHz, where minimal worldwide research exists. A power resolution below 10^−5^ dB in preliminary measurements demonstrated its effectiveness in quantifying signal attenuation due to precipitation across the specified frequencies. Moreover, a strong power law relationship was observed between signal attenuation and precipitation rate for all three frequencies, while, as expected, the higher the frequency, the more pronounced the signal attenuation was.

## 1. Introduction

In recent years, the increasing frequency and intensity of extreme weather events, attributed to climate change, have highlighted the urgent need for accurate and timely precipitation estimations. Precipitation plays a crucial role in various aspects of life, including agriculture, water resource management, and disaster preparedness. The framework of the widely used traditional methods of precipitation estimation, including rain gauges, meteorological satellites, and weather radar systems, although effectual, often faces limitations in providing timely information with high spatial and temporal resolution, particularly in remote or inaccessible regions. In regard to the aforesaid, various cases from the bibliography are reported for easy following, without extensive details (as in cases of systematic reviews), toward the actuality of new approaches, relevant works, and elliptical facts of research in specific frequency bands. As far as the high spatial and temporal resolution of precipitation monitoring, it is very helpful because precipitation exhibits very high spatial and temporal variability, especially over areas with complicated relief [1,2]. Therefore, new approaches must supplement the traditional precipitation measurement methods. The review in [3] discusses various design principles and methods for recording rainfall intensity, and their abilities and limitations. It highlights the potential of contemporary approaches, including signal attenuation in cellular networks and crowdsourced data, to provide more observation sites than conventional methods.

To address these challenges, researchers have turned to innovative approaches that leverage the ubiquitous presence of radio frequency (RF) signals in modern communication systems. By measuring the attenuation of RF signals caused by precipitation, researchers aim to develop reliable methods for estimating precipitation rates in real time. In [4], the impact of heavy rainfall is explored on millimeter-wave propagation and cross-polarization in 5G networks, estimating rainfall attenuation, path loss, and link margin in urban and highway scenarios. The work in [5] presents an experimental study on the combined effects of rain and wind on millimeter wave fixed wireless systems at E-band frequencies, using ITU-R and APT models to estimate attenuation and link budget under tropical conditions. Most studies in this field have concentrated on signals of frequencies exceeding 10 GHz [6]. This preference stems from the fact that measuring precipitation-induced attenuation at lower frequencies presents challenges due to the precipitation’s reduced effect on the signal.

In most cases, the focused studies on frequencies exceeding 10 GHz have utilized data from commercial microwave links (CMLs). The presented work in [7] evaluates the effectiveness of using CMLs for high-resolution precipitation estimation, comparing CML-based precipitation fields with radar, rain gauge, and satellite data and analyzing the influence of link characteristics on estimate reliability. In the Netherlands, numerous CMLs have been employed in various studies for rainfall estimations [8,9,10] and the creation of rainfall maps [11,12]. Similar studies have utilized data from thousands of CMLs for rainfall estimation in Germany [13,14,15]. Such data were also shared between Germany and the Czech Republic for the creation of transboundary rainfall maps between the two countries [16]. Also, over one million 5G stations were used for rainfall estimation in China [17]. In West Africa, several CMLs have served for both rainfall estimations [18] and the generation of rainfall maps [19]. The effectiveness of monitoring urban rainfall through CMLs has also been indicated by a study in France [20]. In [21], a random forest model was used to classify microwave radiometer observations over the Netherlands as dry, shallow, or nonshallow precipitation, demonstrating the model’s effectiveness in detecting shallow precipitation, particularly during summer. Different machine learning models, including statistical and deep learning algorithms, were employed in [22] to predict precipitation using satellite imagery, radar data, and ground-based observations. The evaluation criteria included RMSE, R², and MAE to assess model accuracy, highlighting the effectiveness of machine learning in rainfall prediction. The potential of remote sensing and hybrid models for improved precipitation forecasting was emphasized.

Regarding the limited number of studies focusing on frequencies under 10 GHz, most rely on data from CMLs or signal power measurements from cellular terminals. For example, preliminary rainfall estimations were conducted in West Africa using a 7 GHz CML [23]. In Italy, a cellular terminal was employed for rain classification via the attenuation of a 4G/LTE signal [24]. Rain classification using a cellular terminal operating on the 4G/LTE frequency of 2630 MHz was also conducted via signal power measurements through a customized Android application in Greece [25]. Similarly, cellular terminals were also utilized to study the rain-induced attenuation on the GSM frequency of 1.8 GHz in Taiwan. Similarly, data from cellular terminals operating on 2 GHz were devoted to training a wet/dry classification model in China [26]. In the Philippines, signal power data for the frequency of 5 GHz from SmartBro subscribers were used to create a rain alarm system [27]. Moreover, in Greece, a very accurate experimental setup [28] has been employed for a rain estimation model [29] on 2 GHz. The study presented in [30] demonstrates that microwave links in the 6–8 GHz range can accurately detect rainfall and estimate path-averaged rainfall rates in Seoul, Korea, achieving higher than 80% accuracy (compared to rain gauges).

The prevalence of research in rainfall estimation through signal attenuation is mainly focused on data from CMLs and frequencies higher than 10 GHz. How different temporal sampling strategies affect rainfall intensity estimates using terrestrial microwave links, is discussed in [31], where the impact of sampling intervals and strategies on the accuracy of rainfall measurements is highlighted. It is well known that there are various and severe limitations to the signal data coming from the CMLs, the main ones being the inability to access the data of cellular phone providers, the very long sampling times from 1 to 24 h, the limited measurement accuracy, and the inhomogeneity of the data [6]. To the best of the author’s knowledge, in the international bibliography, there are a few unique experimental setups that measure rain attenuation only at high frequencies above 10 GHz. The transmitter/receiver unit presented in [32] simultaneously operates at 22.235 GHz and 34.8 GHz. Furthermore, the innovative apparatus offered readings based on the antenna reflection coefficient. It employed six separate commercial microwave connection antennas with working frequencies of 18, 22.235, 25.375, 28.500, 34.800, and 38.500 GHz [33]. The work presented in [34] introduces a methodology to model the effects of rain and fog on LiDAR sensor performance, using the Mie scattering theory for simulation-based testing.

In addition to the aforementioned endeavors for alternative rainfall estimation methods, video-based rainfall measurement has emerged as a promising technique too. This method leverages advancements in image processing and computer vision to capture rainfall data with high spatial and temporal resolution. Video-based methods offer the potential for real-time performance and cost-effectiveness, making them a valuable addition to existing rainfall observation networks. Urban surveillance cameras, coupled with advanced deep learning techniques, enable a precise rainfall intensity estimation model developed in a study presented in [35]. Using a hybrid CNN + RNN approach with SimpleRNN, GRU, and LSTM units, the model achieved MAPE values between 3.55% and 6.95%, outperforming traditional methods. This method provides continuous rainfall estimates directly from one-minute videos, offering potential applications in water resource management and flood risk mitigation. The review presented in [36] discusses the development of various algorithms for recognizing raindrops and estimating rainfall from video footage. Despite the advantages of video-based rainfall measurement, challenges such as raindrop visibility, depth of field effects, and motion blur introduce significant errors and uncertainties. Improving algorithm robustness, accuracy, and computational efficiency is essential for the practical application of this method in real-world rainfall monitoring networks.

In brief, this paper’s contribution lies in a novel idea relevant to a test bed comprising our lab-made transmitter-receiver system and all the needed electronic circuits for efficient precipitation measurements operating in frequency bands not thoroughly examined as others in the bibliography. Also, one of the highlights is that the measurements are conducted simultaneously in three different channels, which is very important as it gives a true fusion of three different types of measurements for concluding a more valid result. Furthermore, to prove the well-established measuring system, this is compared to other ones to show its advantages.

In detail, the novelty of our proposed experimental setup lies (1) in the accuracy of the measurement and (2) in the possibility of simultaneous reception and processing of data in three different frequency bands based on a software-based receiver and transmitter. Mainly, this paper introduces a high-precision experimental setup optimized for operation on 2.07, 4.63, and 6.22 GHz frequencies. A similar setup for signal power measurements on frequencies of the 5 GHz region is also presented in [37]. The setup presented herein comprises two systems, a transmitter and a receiver, and offers a power resolution of 0.8 × 10^−5^ dB and a receiving sensitivity of −60 dBm. The purpose of such a setup is to facilitate the development of a rain estimation model based on high-precision signal attenuation measurements and for frequencies that have not been studied extensively, if any, in previous studies. This study makes a dual contribution. Firstly, it provides preliminary measurements of signal attenuation caused by precipitation within a largely non-studied frequency range in this area of expertise. This frequency range is significant because several modern communication protocols operate within it (5G NR, WiFi 6E, LTE, etc.). Secondly, this research distinguishes itself by utilizing a high-precision experimental setup, unlike the typical commercial microwave links with limited measurement accuracy found in most studies.

The rest of this paper is organized as follows: in Section 2, the methodology is presented, in Section 3, the transmitter and the receiver of the experimental setup are described. In Section 4, the study area and datasheet are presented, followed by the results of preliminary power measurements during precipitation events in Section 5. Then, Section 6 includes a discussion, followed by the conclusions (Section 7).

## 2. Methodology

**Figure 1:**
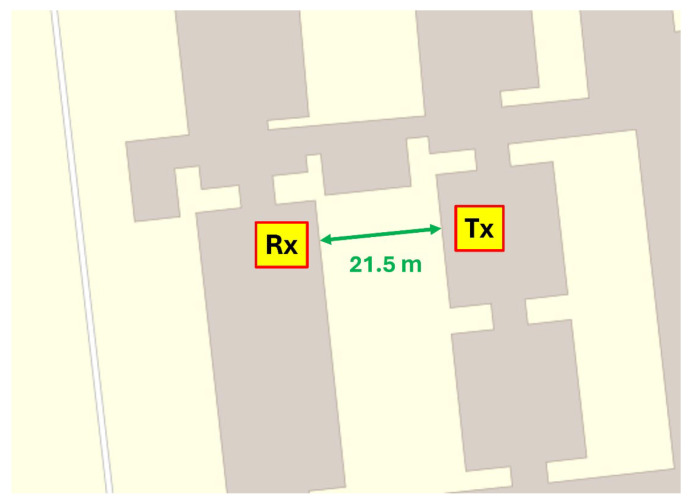
The placement of the transmitter (Tx) and the receiver (Rx) for the conduction of signal power measurements.

The experimental setup was employed for preliminary power measurements during the presence of precipitation, spanning from the latter part of January 2024 to the end of May 2024. The transmitter and the receiver were placed in two adjacent buildings of the Physics Department of the University of Ioannina, as depicted in Figure 1. The separation between them was L = 21.5 m.

For the sake of reproducibility and replicability, the following methodology was followed:

(a) The distance (L) of the microwave link between the transmitter and receiver is such that the rain is spatially uniformly distributed; (b) the transmitted power remains constant; and (c) jitter due to wind and wet antennas is considered negligible as the antennas are in a protected environment.

We define as ${\bar{A}}_{d}$ (in dBm) the average value of the power at the receiver during the time interval Δt, where there is no precipitation before the rain event. The received value of the signal power at time t—in the absence of precipitation—is expressed by A_d_(t) and is affected not only by the intrinsic noise of the electronics but also by other atmospheric parameters such as humidity, fog, and temperature. If the spread of the received power measurements during the time interval Δt has a variance of less than 1%, then the average value is defined as the baseline power A_base_. The signal attenuation during a precipitation event is expressed by A_r_(t) in dBm. The specific rain attenuation in dB/Km for a precipitation event is given by

(1) $$A=\frac{\left|A_{base}-A_{r}\left(t\right){|}_{min}\right|}{\begin{array}{c} L \end{array}}$$

where $A_{r}(t){|}_{min}$ is the minimum signal power during a precipitation event, and L is the microwave link distance in Km. Since each precipitation event corresponds to a rain rate (R) in mm/h, measured by a typical rain gauge, a set of measurements is obtained (R, A).

## 3. The Experimental Setup

The experimental setup discussed in this paper consists of a transmitter and a receiver tailored to operate between 2.07, 4.63, and 6.22 GHz frequencies. The transmitter utilizes voltage-controlled oscillators (VCOs) for signal generation, while the receiver employs two power detectors to measure signal power. This section provides insights into both the transmitter and the receiver, outlining their design and role as part of the complete experimental setup.

**Figure 2:**
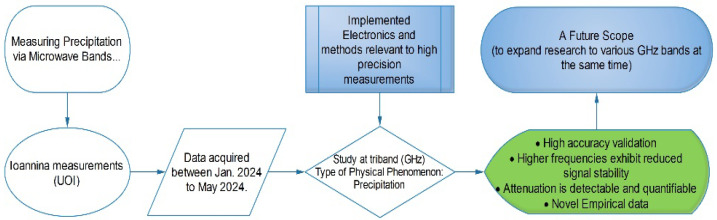
Simple reproducibility plan (SRP).

Specifically, a simple diagram is given for reporting the whole procedure extending from the constructed electronics to the measurements section. This refers to a Simple Reproducibility Plan (SRP), which can also provide a proper understanding of the accomplished procedures. It appears in Figure 2.

### 3.1. The Transmitter

The core of the transmitter system is a Raspberry Pi 4 microcomputer, serving as the central control unit. Transmission across the designated frequencies of 2.07, 4.63, and 6.22 GHz is achieved by three VCOs.

Each VCO’s power supply can be toggled on/off by the microcomputer via manipulating three switches, comprising Bipolar Junction Transistors (BJTs) integrated into a printed circuit board (PCB). Furthermore, the microcomputer dictates the output frequency of each VCO by providing the requisite tuning voltage to it, a process facilitated by a 16-bit Digital-to-Analog Converter (DAC). The DAC is included in the High-Precision AD/DA Board of Raspberry Pi hardware attached on top (HAT).

**Figure 3:**
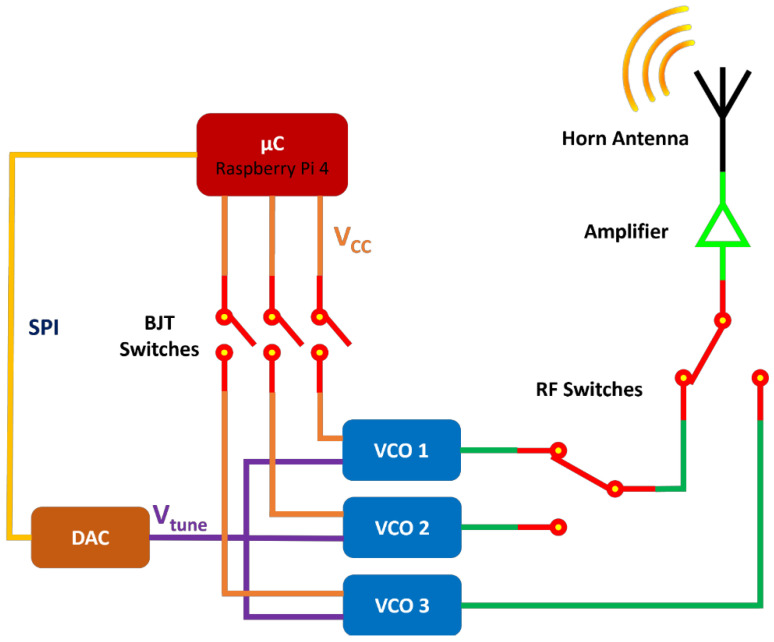
Block diagram of the transmitter system. V_cc_ denotes the supply voltage of each VCO, and V_tune_ is the required tuning voltage that controls the transmission frequency of the VCO.

The outputs from the VCOs connect to a stage that includes two RF switches, the state of which is under the microcontroller’s control. The state of these switches determines which VCO connects to the transmitter’s output. Subsequently, the RF signal levels are increased by an amplifier before being transmitted via a horn antenna. The block diagram of the transmitter appears in Figure 3.

During the operational phase of the transmitter, it initially remains idle, expecting a message sent over the internet by the receiver. That message contains information about the desired transmission frequency, between 2.07, 4.63, and 6.22 GHz. As soon as the message is received, the transmitter’s system processes it to extract the specified frequency, thereafter initializing the appropriate transmission procedures. It selects and then powers on the most suitable of the three VCOs by activating the corresponding BJT switch.

Each BJT switch is controlled by the general-purpose input/output (GPIO) pins of the Raspberry Pi and consists of an NPN transistor at the input stage and a PNP transistor at the output stage. The NPN transistor prevents the current from floating towards the GPIO pins and protects them from potential damage. The power input of the VCO connects to the emitter of the PNP transistor. The remaining two VCOs turn off via the GPIO pins by deactivating the related BJT switches.

With the appropriate VCO now powered, the next step is tuning its transmission frequency by providing the necessary voltage through the DAC. Communication with the DAC succeeds through the serial peripheral interface (SPI) provided by the Raspberry Pi. For transmission at frequencies of 2.07, 4.63, and 6.22 GHz by the VCO 1, VCO 2, and VCO 3, respectively, as denoted in the block diagram of Figure 3, the corresponding tuning voltage is 3.4, 1.8, and 2.9 V. These tunings were conducted by examining the transmission frequency (using a Vector Network Analyzer—VNA) while varying the tuning voltage.

After the output of each VCO, the RF switching stage follows. The first RF switch selects between the output of VCO 1 and VCO 2, while the second selects between the output of the first RF switch and VCO 3, as outlined in Figure 3. Control over the states of these switches is managed through four general-purpose input/output (GPIO) pins, with two allocated for each switch.

The signal exiting the RF switching stage undergoes amplification before eventually reaching the horn antenna and getting transmitted. The transmitter sends a message over the internet to the receiver, informing it that the transmission on the requested frequency is active. It maintains its transmission on the designated frequency until a subsequent message is received from the receiver, prompting a transition to an alternate frequency. This instruction is typically received approximately 1 s after the previous one. Upon receipt of the new instruction, the transmitter initiates the frequency switching process, thereby repeating the operational cycle.

### 3.2. The Receiver

Similar to the transmitter system, the entire operation of the receiver is handled by a Raspberry Pi 4 microcomputer. The receiver’s system comprises a printed circuit board (PCB) housing two RF power detectors, an RF switch for managing signal direction between the power detectors, and two intermediary bandpass filters. These filters exhibit passbands spanning 1960 to 2360 MHz and 4040 to 6500 MHz, respectively.

The receiver utilizes a reconfigurable patch antenna, analyzed in [38]. The antenna incorporates a PIN diode, on which the applied voltage governs the antenna’s frequency response. Consequently, the antenna supports three optimal modes of operation corresponding to voltages of 0, 550, and 740 mV, each optimizing the performance on the frequencies of 2.07, 4.63, and 6.22 GHz, respectively. Also, a 16-bit DAC, akin to the transmitter system, is employed for applying the aforementioned voltages.

The power detectors yield an output voltage linearly proportional to the input signal power. It is measured using a 24-bit Analog-to-Digital Converter (ADC), integrated into the same Raspberry Pi HAT as the DAC. Similarly to the transmitter, the receiver utilizes an amplifier for signal amplification after its reception by the antenna.

The obtained data (by the receiver) is stored both locally in text files and within a database. For this purpose, the Microsoft SQL Server was installed on a remote computer.

**Figure 4:**
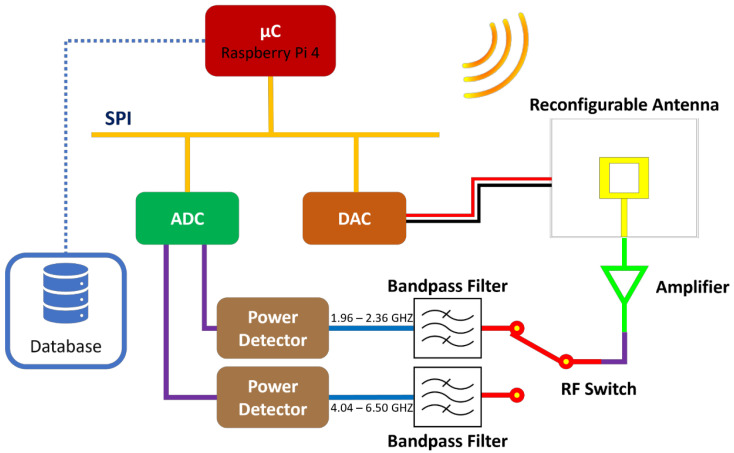
Block diagram of the receiver system.

The receiver’s block diagram is illustrated in Figure 4.

The operational sequence of the receiver commences by sending a message over the internet to the transmitter, soliciting transmission on one of the three specified frequencies: 2.07, 4.63, or 6.22 GHz. Subsequently, the receiver awaits the response message that confirms the active transmission on the desired frequency (by the transmitter).

Upon receipt of the confirmation message, the receiver joins communication via the Serial Peripheral Interface (SPI) with the Digital-to-Analog Converter (DAC), directing it to apply the appropriate voltage to the antenna. This voltage adjustment corresponds to the optimal operational mode of the antenna for the selected frequency.

The transmitted signal is intercepted by the antenna, thereafter amplified, and directed toward the power detector PCB. Each power detector is configured for optimal performance at specific frequencies: one for 2.07 GHz and the other for 4.63 and 6.22 GHz. The receiver, having initiated the signal transmission by the transmitter and thus aware of its frequency, manipulates the state of the RF switch at the input of the PCB using four GPIO pins of the Raspberry Pi. This action directs the signal towards the appropriate power detector, following passage through the respective bandpass filter to mitigate potential interference.

The power detector generates an output voltage (V) proportional to the input signal power (P). Utilizing two channels of the Analog-to-Digital Converter (ADC), the receiver acquires the voltage output from the power detectors. It commands the ADC via the SPI to initiate a voltage reading and subsequently retrieve the result. Utilizing the acquired data, it computes the voltage at the power detector output and, employing the known relationship followed by the power detector, determines the signal power. These data, alongside ADC readings and timestamps, are stored locally on the Raspberry Pi and within the database on the remote computer. When the data are logged, the receiver sends a new message to the transmitter, requesting the transition to a new transmission frequency, and the whole process starts over. The receiver is configured to log data and sends a new message once per second. Consequently, since it cycles between the three frequencies, the sampling time for each frequency is 3 s. An example 20 s timeframe of raw data from 11 March 2024, for the frequency of 2.07 GHz, is included in Table 1.

The receiver’s sensitivity, attributed to the employed power detectors, is −60 dBm. The ADC utilized by the receiver features a 24-bit resolution, with one bit designated for sign representation, thereby offering a practical resolution of 23 bits. In Table 1, The ADC data represent digital outputs from the 23-bit ADC, capturing the amplitude of an analog signal at various time points. Each value is a 23-bit integer corresponding to a specific voltage level of the analog signal. To detect the analog signal, it is digitized by the ADC into 2^23^ possible levels. Generally, after digitization on a 23-bit ADC, each analog voltage level is represented by a value between 0 and 2^23^. Relevant to the ADC configuration employed in the receiver system, it features 22.3 noise-free bits.

The Signal-to-Noise Ratio (SNR) for an ideal ADC is given by the equation [39]:

(2) SNR=6.02b+1.76,

where b represents the number of resolution bits. Given the 22.3 noise-free bits of the ADC, the SNR for the receiver is found 136 dB.

Considering the supported voltage range for the ADC (−5 to +5 V, resulting in a span of 10 V), and factoring in the 22.3 noise-free bits, the resultant voltage resolution stands at 1.9 μV. Moreover, by incorporating the voltage resolution alongside the slope of the linear relationship between input signal power and output voltage on the power detector, one can determine the resolution in signal power equal to 0.8 × 10^−5^ dB.

**Figure 5:**
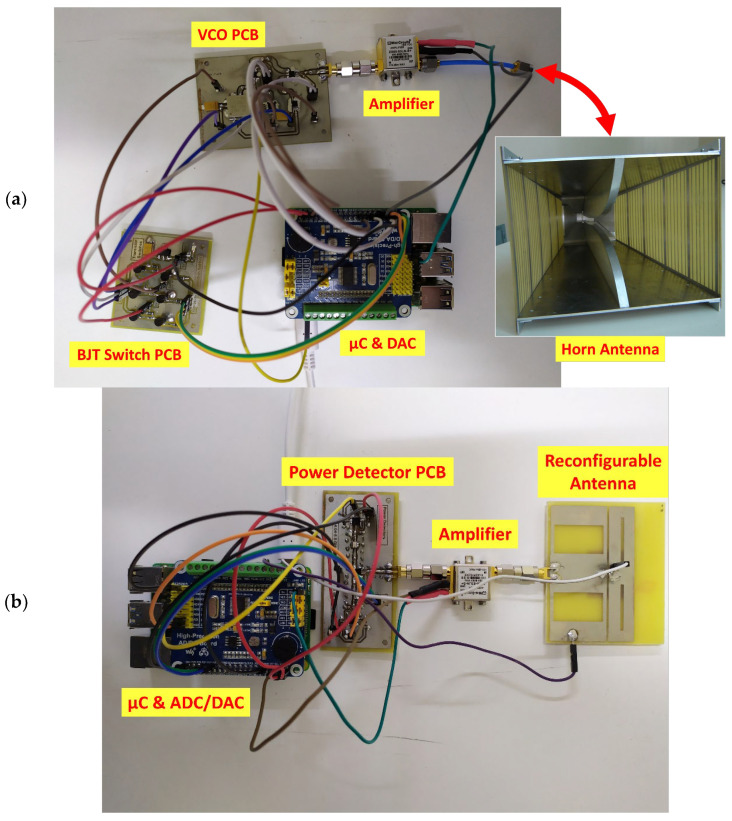
The implemented system of the (**a**) transmitter and (**b**) the receiver under test.

A picture of the implemented transmitter and receiver systems under test follows in Figure 5.

## 4. Study Area and Datasheet

Ioannina city is located in an inland area of Northwestern Greece west of the Pindus mountain range, and its altitude is about 500 m. During the cold period of the year, the region is affected by the Mediterranean cyclones formed over the west and the central Mediterranean Sea and moving eastwards while causing southwesterly airflow, contributing to remarkable precipitation amounts over the west windward areas of Greece. The main type of Ioannina’s precipitation is rain (snow is relatively rare). Also, fog is relatively frequent during anticyclonic conditions, occurring mainly in the early morning. During the warm period of the year, precipitation is caused primarily in the afternoon via the combination of land heating and high static instability. Such conditions are associated with showers and thunderstorms of small duration but high precipitation rates. As it concerns air temperature and absolute humidity, the area experiences the typical Mediterranean seasonal cycle with the highest values in summer and lowest values in winter [40,41]. Precipitation rates for comparison with signal power were obtained from the meteorological station, of the University of Ioannina, which was situated 850 m from the measurement site. These precipitation rates were sampled every 10 min. During the power measurements, a 5 min moving average was computed and applied to the data.

## 5. Results

**Figure 6:**
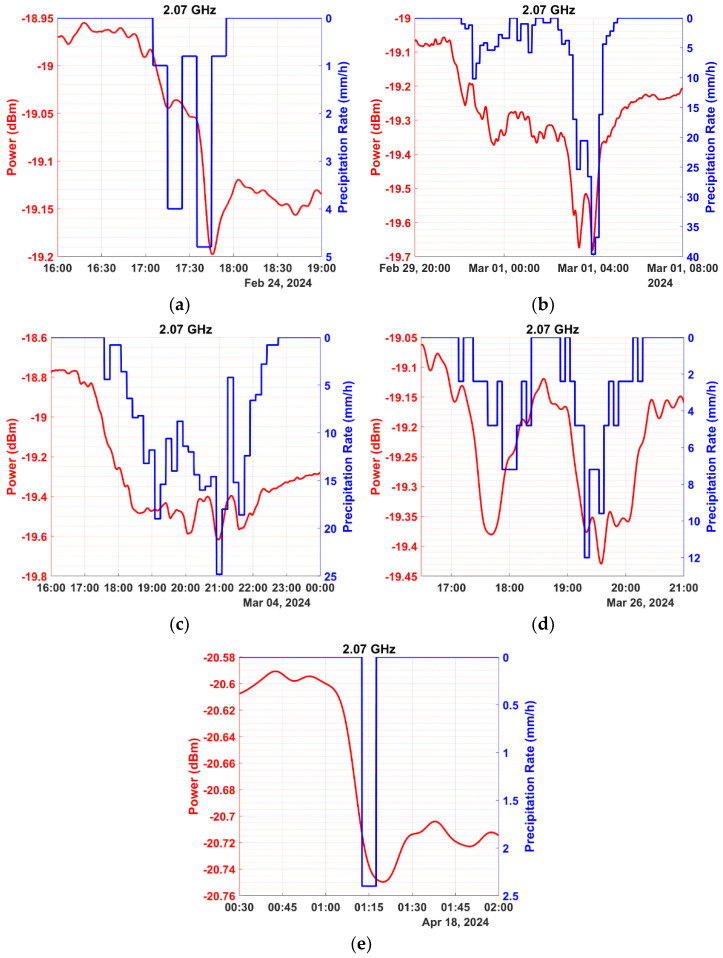
Depiction of instances of five precipitation events (expressed in mm/h, shown in blue) and their corresponding impact on the signal power (expressed in dBm, shown in red) for the frequency of 2.07 GHz and dates: (**a**) 24 February 2024, (**b**) 29 February to 1 March 2024, (**c**) 4 March 2024, (**d**) 26 March 2024, and (**e**) 18 April 2024.

**Figure 7:**
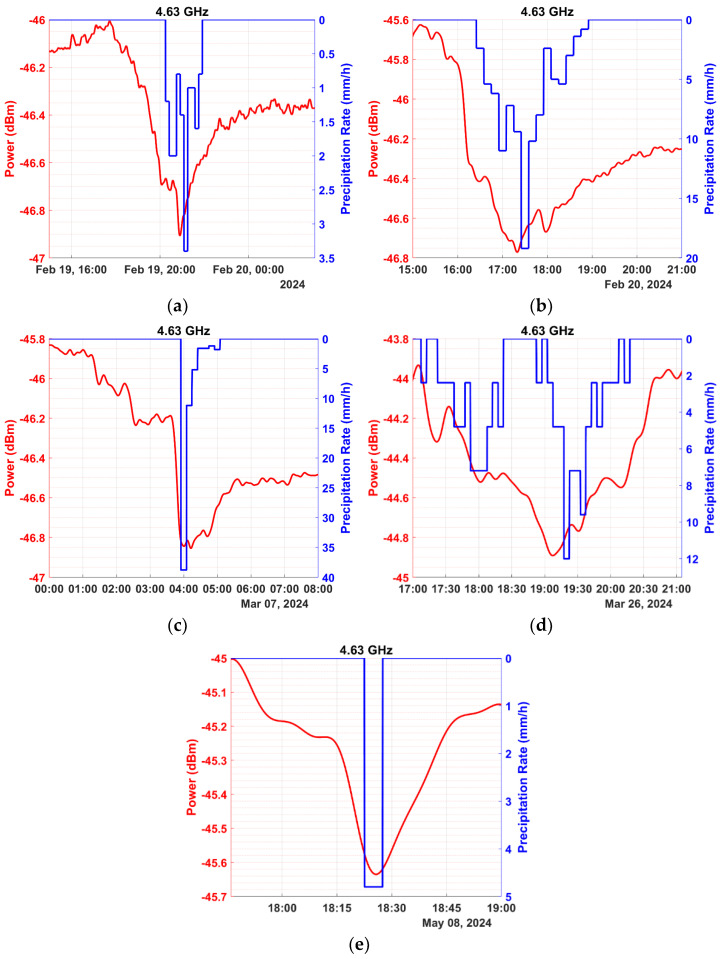
Depiction of instances of five precipitation events (expressed in mm/h, shown in blue) and their corresponding impact on the signal power (expressed in dBm, shown in red) for the frequency of 4.63 GHz and dates: (**a**) 19 February 2024, (**b**) 20 February 2024, (**c**) 7 March 2024, (**d**) 26 March 2024, and (**e**) 8 May 2024.

**Figure 8:**
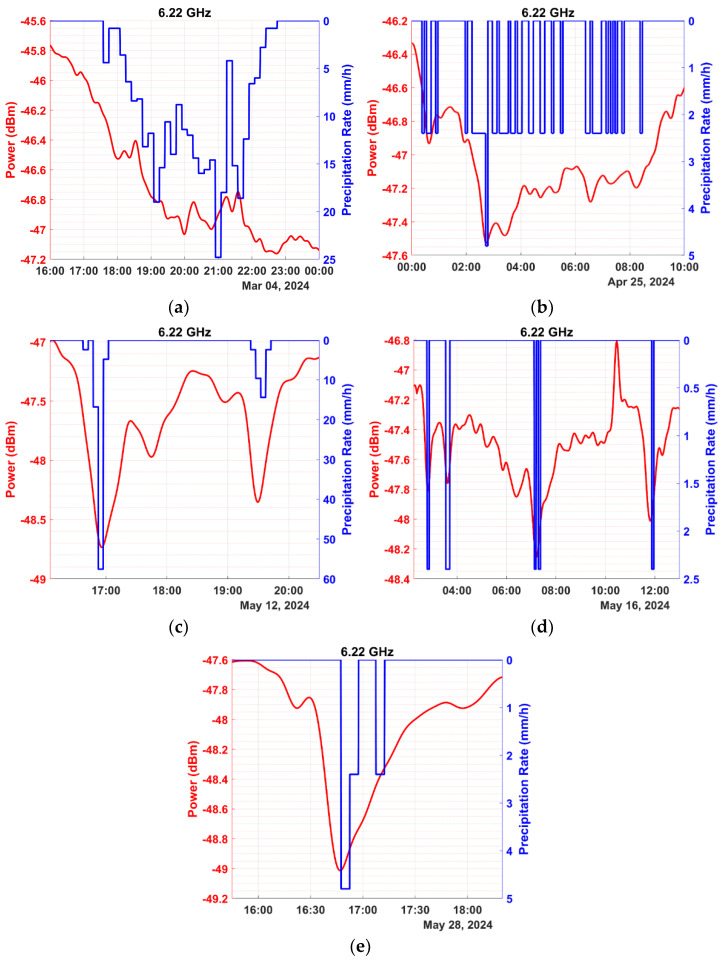
Depiction of instances of five precipitation events (expressed in mm/h, shown in blue) and their corresponding impact on the signal power (expressed in dBm, shown in red) for the frequency of 6.22 GHz and dates: (**a**) 4 March 2024, (**b**) 25 April 2024, (**c**) 12 May 2024, (**d**) 16 May 2024, and (**e**) 28 May 2024.

In this section, we present preliminary results obtained from our special measuring equipment over a period of four months. The data were processed based on the methodology of Section 2. Illustrative instances of power measurements during precipitation events are depicted in the graphs of Figure 6 for the frequency of 2.07 GHz, Figure 7 for 4.63 GHz, and Figure 8 for 6.22 GHz. The precipitation rate axis was inverted, to facilitate comparison with signal power, placing a rate of 0 at the top.

The preceding figures demonstrate the experimental setup’s ability to effectively measure signal attenuation caused by precipitation, even at the 2.07 GHz frequency, where minimal power loss occurs during light precipitation. Conversely, attenuation is more noticeable at frequencies of 4.63 and 6.22 GHz compared to 2.07 GHz, as indicated by the preliminary measurements. Additionally, higher frequencies exhibit reduced signal stability due to shorter wavelengths, which makes the signal susceptible to scattering effects from surrounding obstacles. Nonetheless, attenuation remains detectable and quantifiable in most cases.

It has already been mentioned that the received signal strength (when the precipitation rate is zero) is often referred to as the baseline. The baseline is not constant, since the signal power is affected by interference, noise, multipath propagation, and several other meteorological parameters like water vapor, humidity, and wind, which significantly affect the measurements. Consequently, determining a baseline to serve as a reference when calculating power loss due to precipitation can be challenging. Several studies have endeavored to introduce a method that automates the process of determining the baseline when calculating power loss for each precipitation event [42,43,44,45,46,47,48]. In the present study, the baseline was determined through the methodology presented in Section 2. In cases where the baseline selection needed hard work due to strong signal fluctuations, the corresponding precipitation events were excluded from the analysis. In the example of Figure 6e, the baseline shifts to a lower power level after the precipitation event when compared to its level preceding the event. It is due to the increased humidity lingering in the air after the precipitation is over. While precipitation remains the dominant factor, attenuation due to humidity cannot be overlooked. Even at frequencies as low as 2630 MHz, absolute humidity can cause measurable signal attenuation, though smaller in magnitude compared to that caused by precipitation [25]. It becomes particularly relevant during transitional periods after rainfall, when residual water vapor persists in the atmosphere, and even more so during nighttime, when elevated humidity levels linger for extended periods. It is noted that after a rain event, the atmosphere is enriched with water vapor mainly due to the evaporation of the rain droplets and the ground surface water.

**Figure 9:**
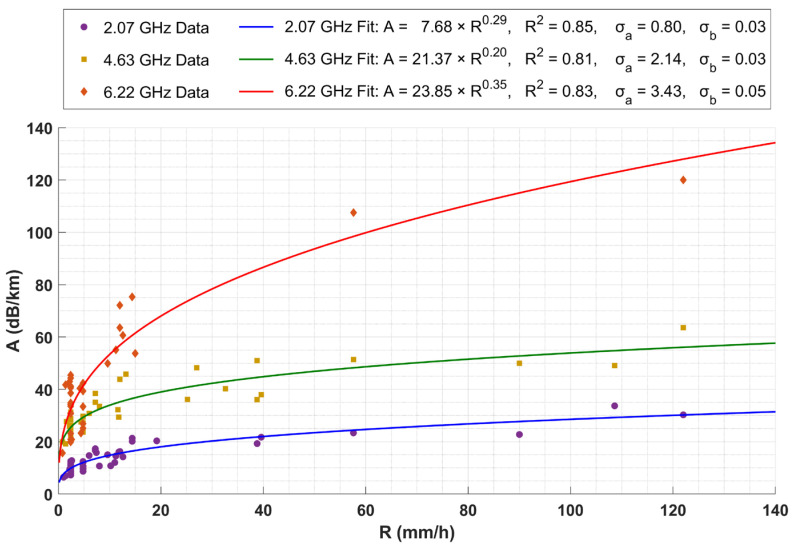
Specific rain attenuation data and corresponding power law fits for frequencies 2.07 GHz and 4.63 GHz.

Following the methodology presented in Section 2, three measurement sets are arising. Each set of measurements follows a power law of the following form between specific rain attenuation (A) in dB/km and rain rate (R) in mm/h, which is consistent with the literature [49]

(3) $$A=aR^{b},$$

where a and b are coefficients dependent on frequency and rain temperature. Multiple precipitation events were inspected, like those presented earlier. The difference between the minimum signal power during a precipitation event and the average signal power throughout a period of minimal signal fluctuations, occurring up to a maximum of Δt = 4 h prior to the event, was calculated to infer signal attenuation. The specific attenuation data for the three frequencies appear in Figure 9. Finally, a fit based on Equation (3) was applied to the data for all three frequencies of 2.07 GHz, 4.63 GHz, and 6.22 GHz. In Figure 9, the presented results display all of the frequencies on common axes.

According to Figure 9, the coefficients a and b defined by the fit results are, respectively, 7.73 and 0.28 for the 2.07 GHz frequency, 21.33 and 0.20 for the 4.63 GHz frequency, and 26.91 and 0.32 for the 6.22 GHz frequency. The corresponding coefficient of determination (R^2^) for each fit is 0.85, 0.81, and 0.83, indicating a strong correlation between attenuation and rain rate in all three cases. Additionally, the results highlight that attenuation increases with frequency, with it being the lowest at 2.07 GHz and the highest at 6.22 GHz, consistent with expectations. As frequency increases, wavelength decreases, drawing nearer to the dimensions of rainwater droplets. It makes the signal more prone to absorption and scattering compared to signals with longer wavelengths.

## 6. Discussion

The presented experimental setup, comprising a transmitter and a receiver, employs high-precision power detectors to ensure accurate signal power measurements and was designed for optimal operation across the frequencies of 2.07, 4.63, and 6.22 GHz. These frequencies were selected due to the limited related research in this region. The experimental setup showed an adequate ability to quantify signal attenuation caused by precipitation for the three frequencies.

Specifically, in more detail, some crucial ideas and points of this work include the following. (a) The experimental work presented in this paper offers novel empirical data in a frequency range not previously extensively explored in previous studies. In similar works, signal attenuation is often converted to specific rain attenuation, expressed as dB/km, to provide a common reference point for generalization over different distances. This conversion, which was also applied in this study, follows a well-established methodology that assumes a consistent precipitation rate, a common practice in the field. (b) The power law used in our research is a well-known empirical model for describing signal attenuation due to precipitation, and its application to this particular frequency range contributes new insights to the field. (c) Regarding the mobility of the transmitter/receiver in terms of different locations, the key factor influencing the results is the line of sight between the transmitter and receiver. As long as this line of sight is maintained, the signal attenuation due to precipitation should not change significantly. (d) This study was designed to isolate and study signal attenuation due to precipitation under controlled line-of-sight conditions. While different locations might introduce variables such as local interference or specific environmental conditions, the primary relationship between precipitation and signal attenuation, as captured by the empirical model, should remain valid. Deviations between the rain rate measured at the weather station and that at the measurement site are inevitable due to the spatial heterogeneity of rainfall, particularly during localized shower events associated with convective clouds (e.g., cumulus and cumulonimbus). However, these deviations have a limited effect on the key findings of this study. At higher rain rates, the attenuation curve exhibits diminishing variation, which reduces the sensitivity of the fitted trend line to small differences in rain rate. As a result, any potential spatial discrepancies are unlikely to significantly affect the fitted curve. For lower rain rate values, which occur more frequently, spatial variability tends to be less pronounced. This is in agreement with the fact that some stratiform clouds (e.g., nimbostratus) are combined with moderate and spatially uniform precipitation rates. Furthermore, the larger volume of data collected at lower rain rates ensures sufficient statistical power to minimize the influence of outliers or instances where the rain rate difference between the two locations was substantial.

Furthermore, given the widespread use of communication devices and protocols operating within this frequency range, any described quantification of signal attenuation due to rainfall could be readily applied in practice with minimal implementation costs, leveraging the existing infrastructure. Preliminary measurements conducted with this setup showed its ability to detect signal attenuation effectively due to precipitation. For all three studied frequencies, a strong power law correlation was observed between the specific rain attenuation and rain rate. As this is a preliminary analysis, we excluded measurements where the baseline could not be clearly defined, according to the definition of the baseline power in the Methodology section. This approach was essential to ensure that only high-quality, reliable data were used to establish the signal attenuation–rain rate relationship. Including measurements with unclear baselines could have exacerbated the uncertainty in the analysis and undermined its credibility.

Potential future directions include the design of similarly high-accuracy setups capable of operating across a broader spectrum of frequencies. Additionally, given the spatial variability of precipitation, optimizing the setup’s location closer to the meteorological station could enhance the accuracy of precipitation rate measurements.

### Comparative Study

A comparative study has the purpose of delving into the dynamics of research works across various and different scales and requirements to find out their operation and common ground from a brief point of view. Consequently, the pursuit is to find at a specific point of abstracting the key elements of each research to be compared efficiently to each other. Based on what has been said previously and the fact that there is a scarcity of data regarding signal power loss due to precipitation in the frequency range studied in this paper, it is impossible to make clear comparisons with the present work. For this reason, we sought rain attenuation measurements for frequency—which is the primary impact attenuation parameter—less than 8 GHz that uses commercial microwave links or cellular terminal networks as measurement setups. Next, some of the few related studies conducted on frequencies close to those of the present study are discussed.

In Burkina Faso, West Africa, signal measurements were recorded every second with a 1 dB precision over a 29 km wireless link at a frequency of 7 GHz [23]. Signal data were assessed using a tipping bucket rain gauge placed along the microwave link path, with measurements taken every five minutes. Data collection occurred from July to September 2012, and high-resolution X-band radar data were also used to correlate the received signal information.

A research team from Ateneo de Manila University utilized the existing 5 GHz Smart Bro fixed wireless network infrastructure in the Philippines [27]. They collected signal levels from over 700 subscribers with updates every minute. Data were gathered from July 2012 to the end of 2013. Rain events were classified using sigma level parameters, and maps with color-coded sigma levels were created to visualize highly intense rainfall events.

In [50], the experiments explored the effect of precipitation on signal attenuation at a frequency of 1.8 GHz, being used by the global system for mobile communications (GSM). Cellular terminals were employed to measure the received signal strength (RSS) from base stations in dBm, with a distance of 400 m between the base station and the mobile device. The experimental results revealed that the attenuation observed during the rainy season was significantly higher than the theoretical predictions and was comparable to the attenuation expected at a frequency of 120 GHz. Additionally, wind was identified as another factor influencing attenuation by causing fluctuations in the RSS levels.

Path-averaged rainfalls were estimated using a microwave network with eight links operating at frequencies between 6 and 8 GHz, covering distances from 5.7 to 37.4 km [30]. The accuracy of estimating rain-induced attenuation on these links exceeded 80%, as verified by simultaneous measurements from rain detectors at weather stations. The signal power resolution was 0.01 dB, and the temporal resolution was 1 min.

In [28], specialized measurement equipment is introduced, with a power resolution of 10⁻⁴ dB and a signal-to-noise ratio (SNR) of 145 dB, with sampling intervals of 0.2 min. Data validation was performed using a standard 0.2 mm per tip rain gauge. The experimental setup featured a transmitter and receiver positioned approximately 20 m apart to ensure uniform rainfall conditions. The antennas were housed in adjacent buildings to avoid errors from wet antenna attenuation and wind jitter. Rainfall measurements ranging from 0.2 to 0.8 mm were collected over a year from 2015 to 2016 on the University of Ioannina campus near Ioannina. Additionally, the same research group developed a rain attenuation model for the lower microwave S-band, validating it with signal and rainfall data from the year-long study [29].

In [24], a team conducted an analysis of rainfall by examining received signal level characteristics from a 4G/LTE cellular terminal. They employed the cellular terminal with the G-Mon application, which provided data on received signal strength and connection parameters. Measurements were taken with a 1 min sampling interval, accumulating over 112 h of data in Catania, Sicily, Italy. The cellular terminal was positioned 200 m from the base station, with a tipping bucket rain gauge placed at the same location for data correlation. Additionally, the researchers developed a classification method using a probabilistic neural network to categorize rainfall into four conditions: no rain, light rain, moderate rain, and heavy rain.

A similar study for the 4G/LTE frequency of 2630 MHz is presented in [25]. A customized Android application was used for signal power measurements using a cellular terminal. The power resolution was 1 dB, and the sampling time was 10 s. The cellular terminal was situated 228m away from the base station. The precipitation rate was classified into five classes, while a classification model showed 88.4% accuracy when predicting a class based on the power loss.

The aforementioned works are presented in Table 2 in comparison with the present study. Table 2 summarizes the measurement method, the frequency of operation, power and temporal resolution, and precipitation estimation. As the analysis shows, all the works have a temporal time resolution of 1 min or less. On the other hand, the comparison demonstrates the outstanding accuracy of our measurement setup and the simultaneous reception in three frequencies based on a software-based receiver and transmitter.

Furthermore, the short path link of a few tens of meters makes it clear that the rain will be uniform and consequently the attenuation and rain rate calibration ideal.

## 7. Conclusions

The study of signal attenuation due to precipitation has garnered significant attention in recent years. While most studies focus on frequencies higher than 10 GHz, owing to the minimal attenuation that signals of lower frequencies undergo, the present study introduced an experimental setup designed specifically for the investigation of attenuation at lower frequencies, namely 2.07, 4.63, and 6.22 GHz. Incorporating a transmitter and a receiver, the setup offers a receiving sensitivity of −60 dBm, as well as high precision in power measurements, with a power resolution of $0.8\times 10^{-5}dB$. Preliminary measurements conducted with this setup demonstrated its efficacy in quantifying attenuation induced by precipitation across the frequencies of 2.07, 4.63, and 6.22 GHz. A first set of data collected over four and a half months revealed that the attenuation is lower at the 2.07 GHz frequency and highest at the 6.22 GHz frequency. A fit applied to the data for the three frequencies indicated the expected power law correlation between specific rain attenuation and rain rate. The contribution of this study is twofold. Firstly, it presents preliminary measurements of signal attenuation due to precipitation in a frequency range that has been minimally explored, if at all, in the existing literature. Several communication protocols operating within this frequency range are prevalent in contemporary applications. Secondly, unlike most studies that use commercial microwave links with limited signal power measurement accuracy, this research employs a high-precision experimental setup. The employed methodology has certain limitations. One is the manual selection of the baseline during the power loss calculation due to precipitation. Future research could focus on developing automated algorithms for more accurate and consistent baseline determination. Additionally, this study was conducted in a specific geographic location with unique topoclimatic conditions, which may affect the generalizability of the results. Further studies should be conducted in various climatic regions to validate and refine the empirical relationships observed in this work. Another limitation is the fixed frequency bands used in the experiment. While the selected frequencies provide valuable insights, exploring a wider range of frequencies in the sub-10 GHz region, where there is a dearth of similar studies, could offer a more comprehensive understanding of signal attenuation due to precipitation.

## Figures and Tables

**Table 1:** Twenty-second timeframe of logged data from 11 March 2024, for the frequency of 2.07 GHz.

ADC Data	Voltage (V)	Power (dBm)	Time
1,589,477	0.947402	−19.228	11 March 2024 12:32:24
1,590,376	0.947938	−19.2499	11 March 2024 12:32:27
1,589,537	0.947438	−19.2294	11 March 2024 12:32:30
1,589,605	0.947479	−19.2311	11 March 2024 12:32:33
1,588,958	0.947093	−19.2153	11 March 2024 12:32:36
1,587,649	0.946313	−19.1833	11 March 2024 12:32:39
1,587,952	0.946493	−19.1907	11 March 2024 12:32:42
1,587,908	0.946467	−19.1896	11 March 2024 12:32:45

**Table 2:** Comparison between the present study and other related works.

Ref	Measurement Method	Frequencies (GHz)	Power Resolution (dB)	Temporal Resolution (min)	Precipitation Estimation
[23]	Commercial Microwave Links	7	1	0.02	Power Law Fit
[27]	Smart Bro Network	5	-	1	Precipitation Classification
[50]	Cellular Terminals	1.8	-	-	Power Law Fit
[30]	Commercial Microwave Links	6–8	0.01	1	Power Law Fit
[28,29]	Customized Setup	2	10^−4^	0.2	Power Law Fit
[24]	Cellular Terminal	4G/LTE	1	1	Precipitation Classification
[25]	Cellular Terminal	4G/LTE (2630 MHz)	1	0.17	Precipitation Classification
This Work	Customized Setup	2.07, 4.63, 6.22	10^−5^	0.05	Power Law Fit

## Data Availability

Data are available upon reasonable request.
